# Clinicopathological characteristics of IgG4-related lung disease

**DOI:** 10.1186/s12890-021-01781-3

**Published:** 2021-12-15

**Authors:** Jia Liu, Yuxiang Liu, Ximing Shen, Zhanghai He, Tingfeng Yu, Li Pang, Xiaoyan Jin, Lingyun Wang

**Affiliations:** 1 Guangdong Provincial Key Laboratory of Malignant Tumor Epigenetics and Gene Regulation and Department of Gastroenterology, Sun Yat-Sen Memorial Hospital, Sun Yat-Sen University, Guangzhou, 510120 China; 2 Department of Radiology, Sun Yat-Sen Memorial Hospital, Sun Yat-Sen University, Guangzhou, 510120 China; 3 Department of Pathology, Sun Yat-Sen Memorial Hospital, Sun Yat-Sen University, Guangzhou, 510120 China; 4 Department of General Practice, Sun Yat-Sen Memorial Hospital, Sun Yat-Sen University, Guangzhou, 510120 China

**Keywords:** Immunoglobulin G4-related lung disease, Immunoglobulin G4-related disease, Clinical characteristics, Pathology, Computed tomography

## Abstract

**Background:**

Immunoglobulin G4-related lung disease (IgG4-RLD) is a rare entity. We retrospectively analyzed the clinical and histopathological characteristics of patients with pathologically confirmed IgG4-RLD to improve the diagnosis rate and reduce the risk of misdiagnosis.

**Methods:**

We screened the pathological reports of 4838 patients with pulmonary surgery and/or biopsy specimens from April 2017 to April 2021 at Sun Yat-Sen Memorial Hospital affiliated with Sun Yat-Sen University, and specimens from 65 patients with suspected IgG4-RLD were subjected to immunohistochemical staining for IgG4 and IgG. Finally, 10 patients with definite IgG4-RLD that was pathologically confirmed were enrolled and analyzed.

**Results:**

The incidence of pathologically confirmed IgG4-RLD was 0.2% (10/4838). The ten patients had an average age of 59.7 years at diagnosis, and the male-to-female ratio was 9:1. The initial clinical manifestations were nonspecific, and cough was the most common symptom (4/10). More than one organ was involved in most patients (8/10), and mediastinal/hilar lymph node involvement was often observed (7/10). Serum IgG4 was analyzed in 6 patients and found to be elevated. Serum tumor marker levels were within the normal range or were slightly elevated. Computed tomography (CT) of the chest and/or ^18^F-fluorodeoxyglucose positron emission tomography-computed tomography (^18^F-FDG PET-CT) imaging revealed that 5 patients had a mixed type, 3 patients had the solid nodular type, and 2 patients had the bronchovascular type. All pulmonary masses and large nodules with solid patterns had spiculated margins and inhomogeneous enhancement with or without pleural indentation and a lobulated appearance. Abundant lymphoplasmacytic cell infiltration and fibrosis were observed in all patients. The expression of IgG4 and IgG was upregulated in the pulmonary sections. Seven patients were treated with glucocorticoids with or without additional immunosuppressants and responded well.

**Conclusions:**

The results of our study suggest that multiple imaging findings, an elevated serum IgG4 concentration, and no significant increase in serum tumor biomarkers could provide diagnostic support for IgG4-RLD, especially for isolated IgG4-RLD or IgG4-RLD that includes other organ involvement that does not aid in establishing the diagnosis.

## Introduction

Immunoglobulin G4-related disease (IgG4-RD) is a recently recognized chronic fibro-inflammatory condition with a histopathological hallmark of dense lymphoplasmacytic cell infiltration, storiform fibrosis and obliterative phlebitis [1]. Almost any organ or tissue can be affected, and these organs include the pancreas, hepatobiliary system, salivary and lacrimal glands, kidney and retroperitoneum [2]. In 2004, Duvic et al. first reported IgG4-related lung disease (IgG4-RLD) [3]. Unfortunately, the literature on IgG4-RLD is limited to single case reports and small case series. Moreover, most of these cases were not pathologically confirmed by lung tissue. Other causes, such as pulmonary infection and congestion, may also lead to pulmonary lesions similar to IgG4-RD, which may be mistaken for IgG4-RLD. Thus, the diagnosis of IgG4-RLD is very challenging, especially with isolated lung involvement or other anatomical site involvement that does not help establish the diagnosis, such as pituitary, meninge and nasal sinus involvement. In this case, IgG4-RLD is often confirmed incidentally from the histopathological findings of lung biopsy or from surgery due to a suspected pulmonary malignancy. Therefore, the clinical analysis of IgG4-RLD based on pathological diagnosis may lead to a more accurate understanding of the disease and improve the diagnosis of this entity. We retrospectively analyzed the clinicopathological characteristics of patients with IgG4-RLD, which was verified pathologically to reduce the risk of misdiagnosis.

## Methods

### Patients

**Fig. 1:**
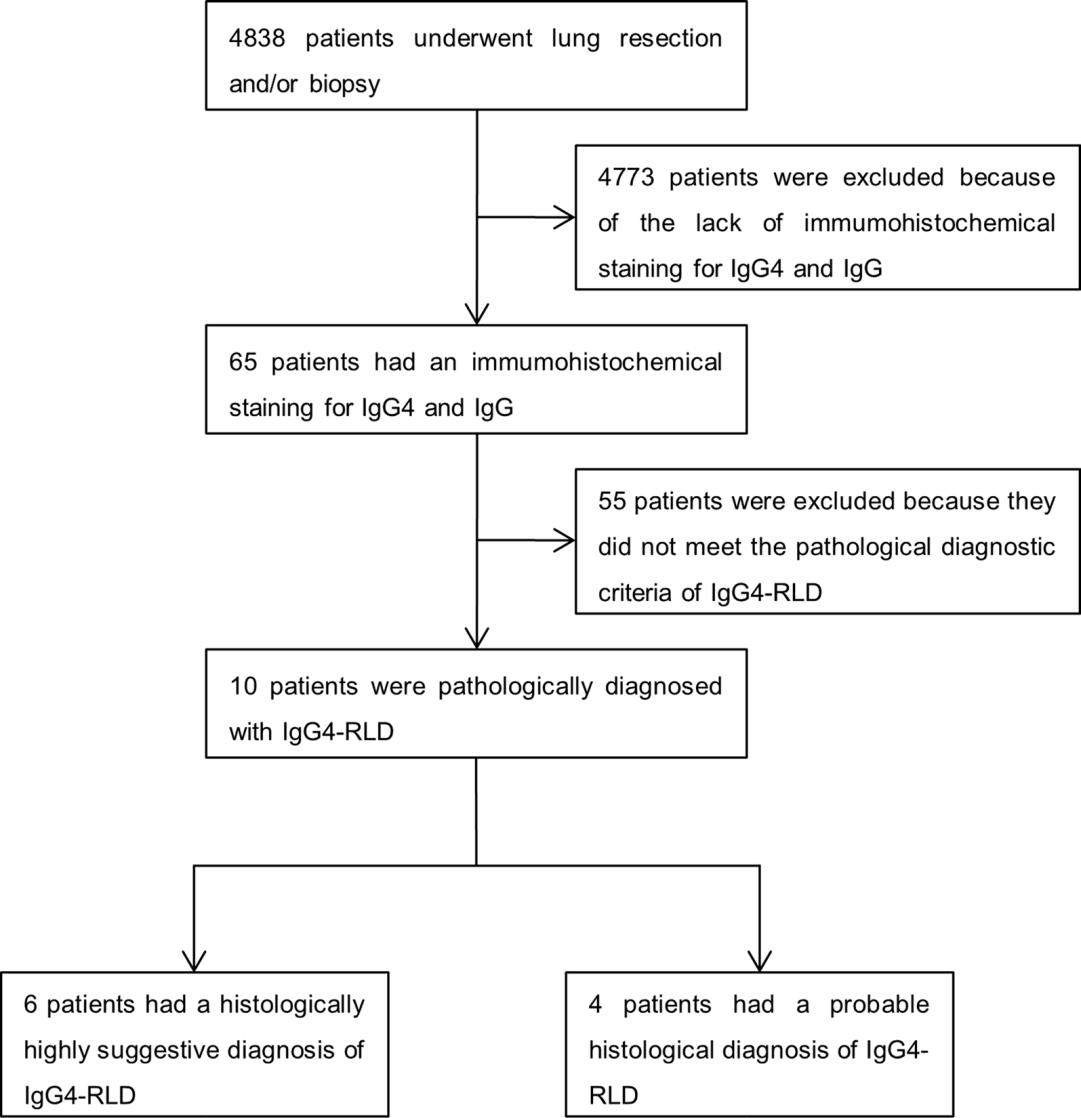
Flow diagram of the screening and diagnostic process for IgG4-related lung disease

This retrospective study was conducted at Sun Yat-Sen Memorial Hospital affiliated with Sun Yat-Sen University. We reviewed the pathological reports of 4838 patients who underwent biopsy and/or lung resection between April 2017 and April 2021, and we used the electronic pathological record system to obtain these data. The levels of IgG4 and IgG were assessed by immunohistochemical staining of lung tissue specimens from 65 patients with suspected IgG4-RLD. According to the 2012 international pathological consensus of IgG4-RD [4], which is described briefly below, a total of 10 patients were ultimately pathologically diagnosed with IgG4-RLD, of which 6 patients had pathological results that were highly suggestive of an IgG4-RLD diagnosis and 4 patients had a probable histopathological diagnosis of IgG4-RLD. The flow diagram of the screening and diagnostic process is presented in Fig. 1. Of the 10 patients, 4 patients (cases 1–4) underwent pulmonary lobectomy via video-assisted thoracoscopic surgery (VATS) based on suspicion of lung cancer, 1 patient (case 5) underwent open lung biopsy (OLB), 1 patient (case 6) underwent percutaneous lung biopsy (PLB) and 4 patients (cases 7–10) underwent transbronchial lung biopsy (TLB). Patients with malignant tumors, lymphoproliferative disorders, Castleman’s disease and sarcoidosis were excluded. Once the patients were diagnosed, relevant clinical data were extracted from the electronic medical records. The clinical, serological, radiological and histopathological features, treatments administered and therapeutic response of the patients were retrospectively summarized and analyzed. The present study was performed according to the tenets outlined in the Declaration of Helsinki and was approved by the Ethics Committee of Sun Yat-Sen Memorial Hospital, Sun Yat-Sen University (number, SYSEC-KY-KS-2021-225). The study was exempt from obtaining informed consent because of its retrospective nature.

## Diagnostic criteria of IgG4-RLD based on histopathology

The histopathological diagnostic criteria were based on the 2012 international pathological consensus of IgG4-RD [4], which are based primarily on histopathological features such as abundant lymphoplasmacytic cell infiltration, fibrosis (usually storiform fibrosis) and obliterative phlebitis. The number of IgG4 + plasma cells and the IgG4 + /IgG + plasma cell ratio in the tissues are of secondary importance. Three categories were proposed for the pathological diagnosis of IgG4-RLD: histopathologically highly suggestive, histopathologically probable and histopathologically insufficient. The criteria for each category are listed below. Patients in histopathologically highly suggestive and histopathologically probable categories were included in the current study.

1. Histopathologically highly suggestive criteria:
  1.  ≥ 2 items from the list of histopathological features.
  2. The number of IgG4 + plasma cells was > 50 cells/high-power field (HPF).
  3. An IgG4 + /IgG + plasma cell ratio > 40%.

2. Histopathologically probable criteria:
  1. At least 1 item from the list of histopathological features.
  2. The number of IgG4 + plasma cells was > 50 cells/HPF from the lung surgical specimens, > 20 cells/HPF from the biopsy, or an IgG4 + /IgG + plasma cell ratio > 40%.
  3. Additional evidence: A serum IgG4 concentration > 2.01 g/l according to the detected range of our hospital or other organ involvement proven by imaging or histopathological examinations.

3. Histopathologically insufficient criterion: Any case that does not conform to the above two categories.

### Histopathological studies

Hematoxylin and eosin (H&E) staining and immunohistochemical staining for IgG4 and IgG were conducted at the Department of Pathology in our hospital. Two pathologists (Ximing Shen and Zhanghai He) independently reassessed the lung tissue sections. The sections that had immunohistochemical staining for IgG4 and IgG were observed under low-power microscopic magnification (× 40) to identify three different hot spots of IgG4 + plasma cells. Then, the numbers of IgG4 + plasma cells were determined in the three fields using HPF counts (× 400), and the average was taken for each investigator. The same three fields were used to count the number of IgG + plasma cells, and then the IgG4 + /IgG + plasma cell ratio in the plasma cells was calculated. The numbers obtained from the two researchers were averaged, and this average was presented as the final result. However, when significantly different counts for a section were obtained, a double-headed microscope was used to reach an agreement.

### Imaging

All included patients underwent thoracic computed tomography (CT) and/or ^18^F-fluorodeoxyglucose positron emission tomography-computed tomography (^18^F-FDG PET-CT) imaging at baseline. A professional radiologist (Yuxiang Liu) carefully reviewed these images. According to the predominant radiological abnormality on CT examination, the patients were divided into groups based on five types as mainly proposed by Inoue and colleagues: solid nodular, alveolar consolidative, round ground-glass opacity (GGO), bronchovascular and alveolar interstitial [5]. The solid nodular type is characterized by a solitary nodule or a mass; the round GGO type is characterized by multiple round-shaped GGOs; the bronchovascular type is characterized by bronchovascular bundle and interlobular septa thickening; the alveolar interstitial type is characterized by bronchiectasis, honeycombing and diffuse GGOs; and the alveolar consolidative type is characterized by segmental or lobar airspace consolidation [6–8]. When two or more types coexisted, they were classified as having a mixed type. The diameters of pulmonary solid nodules and masses were defined as follows: a small nodule was defined as having a diameter < 1 cm; a large nodule was defined as 1 cm ≤ diameter ≤ 3 cm; and a mass was defined as having a diameter > 3 cm [9].

### Follow‑up

The follow-up period ended in October 2021. All 10 patients with IgG4-RLD completed follow-up, with an average of 27 months (range 6–54 months). The clinical, serological and imaging data of these patients were documented in detail at each follow-up visit.

## Results

### Clinical and serological features

The clinical and serological features of the 10 patients with IgG4-RLD are displayed in Table 1. The mean age of the patients at diagnosis was 59.7 years (range 50–67 years), and the male-to-female ratio was 9:1. The time from onset to diagnosis averaged 26.5 months (range 0.2–120.0 months). Of the 10 patients, 1 patient had concomitant chronic obstructive pulmonary disease, and 3 patients had a history of tuberculosis that had been cured. In addition, 6 patients had a history of smoking. The most common initial symptom was cough (4/10, 40%). Other symptoms included chest or back pain (2/10, 20%), hemoptysis (2/10, 20%), low-grade fever (1/10, 10%) and ocular symptoms (1/10, 10%). Three patients were initially asymptomatic and were incidentally identified as IgG4-RLD by abnormal pulmonary changes on chest radiological examinations (3/10, 30%). The majority of the patients (8/10, 80%) had multiple organs involved (mean number 2.4, range 1–6). Mediastinal/hilar lymph nodes were the most commonly involved extrapulmonary organs (7/10, 70%), followed by the nasal sinus (3/10, 30%), lacrimal gland (1/10, 10%), parotid gland (1/10, 10%), submandibular gland (1/10, 10%) and pituitary gland (1/10, 10%). Serum IgG4 was found to be elevated in 6 patients (> 2.01 g/l), while 4 patients did not take the test because of discharge from the hospital. The serum erythrocyte sedimentation rate (ESR) level was elevated in 9 of the 10 patients (> 15 mm/h), and the high-sensitivity C-reactive protein (hs-CRP) level was elevated in 6 of the 10 patients (> 3 mg/l). Of the 10 patients, 3 patients had a slight increase in serum cytokeratin fraction 21-1 (CYFRA 21-1) levels (> 3.3 ng/ml). One patient had a slightly elevated concentration of serum neuron-specific enolase (NSE) (> 16.3 ng/ml), while the serum carcinoembryonic antigen (CEA) and cancer antigen 125 (CA125) concentrations of all of the patients were within the normal range.

**Table 1:** Clinicopathological features of 10 patients with IgG4-related lung disease

Case No	Age (years)	Sex	Clinical features		Serological findings							Diagnostic technique	Histopathological examination		
			Initial symptoms	OOI	IgG4 (g/l)	ESR (mm/h)	hs-CRP (mg/l)	NSE (ng/ml)	CYFRA 21-1 (ng/ml)	CEA (ng/ml)	CA125 (U/ml)		IgG4 + (/HPF)	IgG + (/HPF)	IgG4 + /IgG + (%)
1	65	F	No	No	NA	8	1.2	17.2	3.1	1.9	10.6	VATS	97	214	45.3
2	50	M	No	Lymph node	NA	20	0.3	10.4	3.5	1.9	6.9	VATS	78	175	44.6
3	58	M	Cough, hemoptysis	No	NA	18	1.0	10.7	4.1	1.5	1.5	VATS	52	130	40.0
4	64	M	Chest pain	Nasal sinus	NA	61	9.5	8.4	3.8	2.4	29.3	VATS	56	129	43.4
5	58	M	Cough, low-grade fever	Lymph node	4.6	101	156.8	9.3	1.8	1.4	13.3	OLB	65	151	43.0
6	58	M	Back pain	Lymph node, nasal sinus	11.7	95	117.3	7.7	2.5	2.5	10.1	PLB	29	112	25.9
7	64	M	No	Lymph node, nasal sinus	3.3	77	49.7	11.0	1.7	1.2	9.5	TLB	43	195	22.1
8	56	M	Cough, hemoptysis	Lymph node	8.6	52	23.3	10.7	2.7	1.1	10.0	TLB	122	248	49.2
9	67	M	Cough	Lymph node	7.3	87	25.8	10.6	2.3	0.9	18.7	TLB	34	113	30.1
10	57	M	Ocular symptoms	Lymph node, pituitary, lacrimal, parotid and submandibular gland	56.3	92	3.0	8.2	2.1	0.7	17.3	TLB	40	62	64.5

Normal ranges are as follows: *IgG4* 0.03–2.01 g/l, *ESR* 0–15 mm/h, *hs-CRP* 0–3 mg/l, *NSE* ≤ 16.3 ng/ml, *CYFRA 21-1* ≤ 3.3 ng/ml, *CEA* ≤ 5 ng/ml, *CA125* ≤ 35 U/ml. *OOI* other organ involvement, *IgG4* immunoglobulin G4, *ESR* erythrocyte sedimentation rate, *hs-CRP* high-sensitivity C-reactive protein, *NSE* neuron-specific enolase, *CYFRA 21-1* cytokeratin fraction 21-1, *CEA* carcinoembryonic antigen, *CA125* cancer antigen 125, *HPF* high-power field, *IgG* immunoglobulin G, *F* female, *M* male, *NA* data not available, *VATS* video-assisted thoracoscopic surgery, *OLB* open lung biopsy, *PLB* percutaneous lung biopsy, *TLB* transbronchial lung biopsy

### Imaging characteristics

**Fig. 2:**
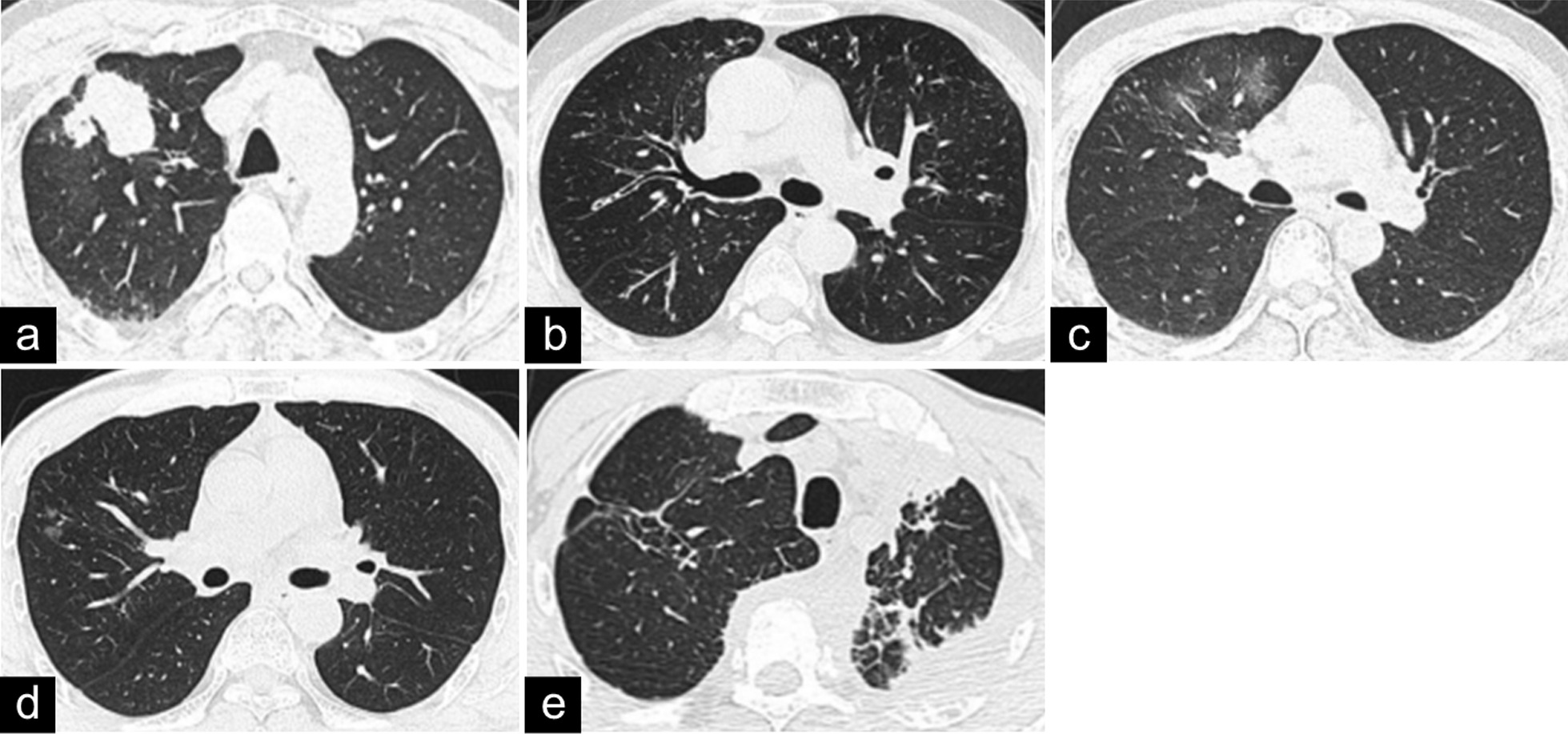
Radiological manifestations of IgG4-related lung disease on chest computed tomography. **a** A mass with a spiculated margin and pleural indentation can be observed in the right lung. **b** Thickening of the bronchovascular bundles and bronchiectasis are noted. **c** Diffuse ground-glass opacities can be observed in the right lung. **d** Round-shaped ground-glass opacities can be observed in the right lung. **e** Alveolar consolidation and thickening of the interlobular septa can be observed in the left lung

The pulmonary imaging characteristics of the 10 patients are summarized in Table 2. Representative images are shown in Fig. 2. Thoracic CT and/or ^18^F-FDG PET-CT findings revealed that 5 patients had a mixed type, 3 patients had the solid nodular type, and 2 patients had the bronchovascular type. Eight patients had pulmonary masses and/or nodules with solid patterns. Of these 8 patients, 3 patients had a single lung mass, and 5 patients had large lung nodules, while all of the patients had multiple small nodules mostly scattered in all of the lung lobes. In addition to a lobulated shape in 1 patient and pleural indentation in 4 patients, these pulmonary masses and large nodules had spiculated margins and heterogeneous densities. The pulmonary nodules were bilateral in 6 patients, while 2 patients had lesions confined to the right lung. The features of the masses or nodules of the 8 patients are summarized in Table 3. Thickening of the bronchovascular bundles, thickening of the interlobular septa, round-shaped GGOs, diffuse GGOs, bronchiectasis, alveolar consolidation, thickening of the pleura and enlarged mediastinal/hilar lymph nodes were observed in 5, 1, 2, 1, 4, 1, 4 and 7 patients, respectively. Nodular calcifications occurred in only 1 patient. Honeycombing, pleural fusion and cysts were not observed in any of the patients.

**Table 2:** Imaging characteristics of 10 patients with IgG4-related lung disease

Case No	Mass/nodule	Thickening of the bronchovascular bundles	Thickening of the interlobular septa	Round-shaped GGOs	Diffuse GGOs	Bronchiectasis	Alveolar consolidation	Thickening of the pleura	Enlarged mediastinal /hilar lymph nodes	Type
1	+									Solid nodular
2	+	+		+		+			+	Mixed
3	+	+				+				Mixed
4	+	+		+		+		+		Mixed
5	+								+	Solid nodular
6	+		+				+	+	+	Mixed
7	+								+	Solid nodular
8	+				+				+	Mixed
9		+						+	+	Bronchovascular
10		+				+		+	+	Bronchovascular

*GGO* ground-glass opacity

**Table 3:** Imaging features of the pulmonary masses or nodules in 8 patients with IgG4-related lung disease

Case No	Mass	Large nodule	Small nodule	Solid pattern	Spiculated margin	Lobulated shape	Pleural indentation	Heterogeneous enhancement	Location
1		+	+	+	+			+	All lobes
2		+	+	+	+			+	Right upper and lower lobe
3		+	+	+	+		+	+	All lobes
4	+		+	+	+		+	+	All lobes
5	+		+	+	+			+	All lobes
6		+	+	+	+			+	All lobes
7		+	+	+	+		+	+	All lobes
8	+			+	+	+	+	+	Right upper lobe

### Pathological findings

**Fig. 3:**
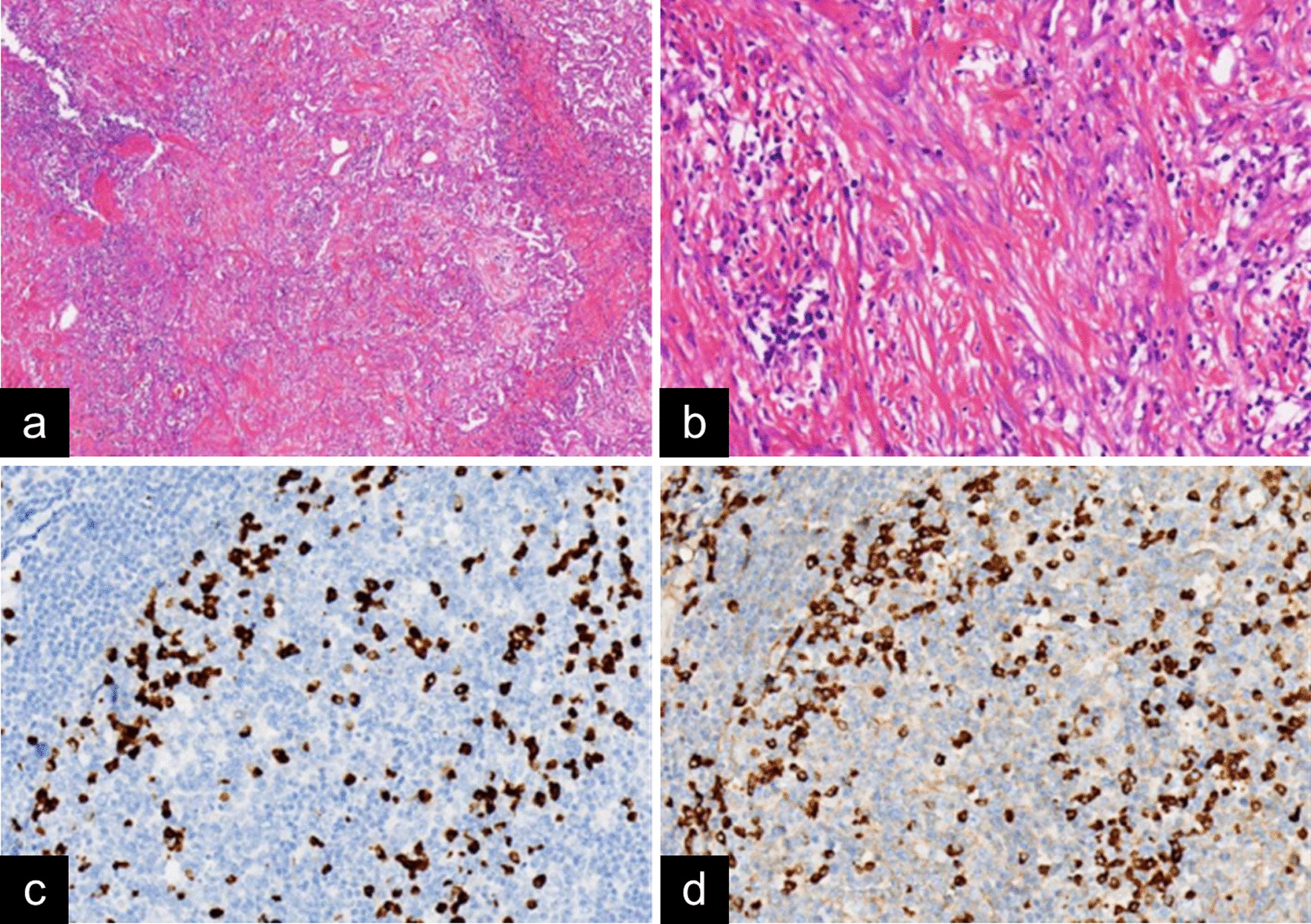
Histopathological findings of IgG4-related lung disease. **a** Dense lymphoplasmacytic cell infiltration and storiform fibrosis can be observed at low magnification (H&E; × 40). **b** Abundant lymphoplasmacytic cells and fibrosis can be observed at high magnification (H&E; × 200). **c** Numerous IgG4 + plasma cells are shown (IgG4 immunostaining; × 200). **d** Numerous IgG + plasma cells are shown (IgG immunostaining; × 200)

The incidence of pathologically confirmed IgG4-RLD was 0.2% (10/4838). All of the lung tissue specimens from the 10 patients with histopathologically confirmed IgG4-RLD were available. Most of the lung specimens appeared gray-white or gray-yellow in color. H&E staining revealed chronic pulmonary inflammation: dense lymphoplasmacytic cell infiltration (10/10, 100%) and fibrosis (10/10, 100%). Representative images are shown in Fig. 3. No malignancy or dysplasia was found in any patients with IgG4-RLD. The immunohistochemistry staining sections for IgG4 and IgG of the 10 patients were carefully reviewed, and the results demonstrated that IgG4 and IgG were highly expressed in plasma cells in IgG4-RLD sections. Meanwhile, the IgG4 + /IgG + plasma cell ratio was determined in the lung tissues of patients with IgG4-RLD. The results are shown in Table 1, and representative images are shown in Fig. 3.

### Therapies administered and the therapeutic response

Seven patients were treated with glucocorticoids with or without other immunosuppressants; 5 patients were treated with prednisone only, and 2 patients were treated with prednisone combined with cyclophosphamide. Two patients underwent lung surgery and did not take prednisone or immunosuppressive drugs. One patient with mild disease received only symptomatic treatment. The symptoms improved in all of the patients after treatment and before they were discharged from the hospital. All of the patients completed several follow-up visits as required. The serum IgG4 level decreased gradually in the 6 patients who had initially elevated serum IgG4. However, in 1 patient, the serum IgG4 level increased again when the prednisone dosage was reduced. Follow-up chest CT was performed in all patients. Six patients showed improvement on chest CT, with nodule or mass shrinkage. Two patients showed no changes in the size of pulmonary nodules or the extent of bronchovascular bundle thickening. Two patients relapsed and presented new pulmonary nodules, but these nodules disappeared after restarting prednisone therapy combined with additional immunosuppressive drugs.

## Discussion

IgG4-RLD is a rare condition that is not well understood, and the incidence of IgG4-RLD is unknown. Our study showed that the incidence of pathologically confirmed IgG4-RLD was 0.2% in our hospital. Herein, we analyze and report the clinicopathological characteristics of the 10 patients with pathologically confirmed IgG4-RLD based on a single-institution experience.

In our study, there were several important findings: (1) Elevated serum IgG4 was observed for all patients in whom it was measured, and there were no significant elevations in serum tumor markers in the patients with IgG4-RLD. (2) The clinical manifestations and radiological findings were nonspecific. Some patients were initially asymptomatic; the pulmonary lesions in these patients were incidentally identified on chest imaging examinations. Moreover, because the results on imaging often mimic pulmonary malignancy, patients with IgG4-RLD might be misdiagnosed with lung cancer and undergo unnecessary lung resection. The involvement of other organs, as demonstrated by radiological or histopathological findings, can also be helpful for diagnosis. However, when the lung is independently affected or there are lung lesions in combination with the involvement of other anatomical sites that do not help establish a definite diagnosis, the diagnosis of IgG4-RLD is very challenging. Thus, we suggest a brief diagnostic strategy to distinguish IgG4-RLD from lung carcinoma: multiple findings on imaging, an increase in serum IgG4, and no significant increase in serum tumor biomarkers. (3) Most patients responded well to prednisone with or without other immunosuppressants.

The clinical symptoms of IgG4-RLD are nonspecific and mainly depend on the location of the pulmonary lesions. Our study showed that the initial respiratory symptoms were cough, chest or back pain, hemoptysis and low-grade fever. Pulmonary abnormalities were occasionally found by chest radiological examinations in asymptomatic patients. This phenomenon has also been reported in previous studies to various extents [5–11].

In 2009, Inoue and colleagues proposed four major types based on the predominant radiological abnormality: solid nodular, round-shaped GGO, bronchovascular and alveolar interstitial [5]. In addition, an additional alveolar consolidation type was observed as the fifth type of imaging classification [6–8]. In the present study, pulmonary masses and nodules were commonly observed, and notably, most were associated with the manifestations of other types, such as bronchovascular bundle thickening, bronchiectasis, interlobular septa thickening, round-shaped GGOs, diffuse GGOs and consolidation. It was therefore not easy to classify them as one of the above five types. We divided these patients into mixed types when two or more types occurred together. Moreover, we found that all pulmonary masses and large nodules with a solid pattern had spiculated margins and inhomogeneous enhancement with or without pleural indentation and a lobulated appearance, and these characteristics were usually indicative of malignancy [12, 13]. These overlapping characteristics of IgG4-RLD and lung cancer on CT suggest that these two diseases may share some pathological mechanisms. For lung cancer, the appearance can be explained by the contraction of internal fibrosis, different growth rates of cancerous cells and the extension of malignant cells along the interstitium [14]. Inoue et al. demonstrated that pathological findings such as fibrosis and diffuse lymphoplasmacytic cell infiltration along the interlobular septa corresponded to the radiological findings of IgG4-RLD [5], which was also observed in our study. We speculate that the imaging manifestations of IgG4-RLD that resemble lung cancer might result from shared characteristics, such as fibrosis and dense lymphoplasmacytic cell infiltration, which mimics the pattern of cancerous invasion. Thus, it is a great challenge to differentiate IgG4-RLD with a pulmonary mass and/or large nodules from lung cancer.

To meet this challenge, we should focus on the following points, especially when patients present with only lung involvement or other organ involvement that does not help establish a definitive diagnosis: (1) In addition to pulmonary masses and/or large nodules, attention should be given to the radiological manifestations of the other subtypes, as mentioned above, which may offer some support for a diagnosis of IgG4-RLD. (2) In this case, serological testing for IgG4 should be performed. Serum IgG4 has high diagnostic value for IgG4-RD, and its concentration has a positive correlation with the number of organs involved [15]. Wang and colleagues reported that 84% of patients with IgG4-RLD had an elevated serum IgG4 concentration [16]. (3) In patients who were initially suspected of having lung cancer, the levels of traditional serum biomarkers are usually measured in routine examinations for the early diagnosis of pulmonary malignancy. Some studies have demonstrated the importance of serum biomarkers in the diagnosis of pulmonary malignancy [17–20]. They revealed that the concentrations of serum biomarkers such as NSE, CYFRA 21-1, CEA, and CA125 are significantly higher in lung cancer than in pulmonary benign diseases, and various biomarker panels were found to increase the sensitivity and improve the accuracy of the diagnosis of lung cancer. Our study showed that all patients with IgG4-RLD had normal concentrations of serum biomarkers or only a slight increase, while serum IgG4 was elevated in all patients in whom it was measured. However, serum IgG4 is not routinely examined in many hospitals until now. It is not economically feasible to measure serum IgG4 in all patients with pulmonary abnormalities. Therefore, for patients who are initially suspected of having lung carcinoma, if the imaging findings show several different manifestations of lung lesions, such as masses or nodules, bronchovascular bundle thickening, interlobular septa thickening, pleural thickening, bronchiectasis, round-shaped GGOs, diffuse GGOs and consolidation, and the concentrations of NSE, CYFRA 21-1, CEA, and CA125 are within normal limits or are slightly elevated, the serological level of IgG4 should be detected. An increase in serum IgG4 can offer diagnostic support for IgG4-RLD, and experimental steroid therapy can be administered to further confirm the diagnosis. If serum IgG4 is not elevated and lung carcinoma is strongly suspected, lung biopsy can be used to establish the diagnosis. Interestingly, our study showed that ESR and hs-CRP were also elevated in most of the patients, which might reflect acute inflammation in IgG4-RLD.

Glucocorticoids are commonly used as the first-line treatment of IgG4-RD, and most patients respond well to this intervention. However, there is currently no universal consensus on the duration and tapering regimens of glucocorticoids. Glucocorticoids can be combined with immunosuppressive drugs as a first-line treatment when there are factors for recurrence, such as multiple organ involvement, elevated serum IgG4 and immunoglobulin E levels and increased eosinophil counts in the peripheral blood [21]. Consistent with previous reports [7, 9], our study demonstrated the effectiveness of glucocorticoids with or without immunosuppressive drugs for the treatment of IgG4-RLD. Retreatment with glucocorticoids in combination with immunosuppressive drugs still allowed for significant improvement in the patients who experienced relapse. Up to the last follow-up, 1 patient who received symptomatic therapy only remained in a stable condition, as was reported in Sun’s study [7]. This indicates that some patients might have self-limiting IgG4-RLD. However, little is known about the natural history of IgG4-RLD because the follow-up durations in these studies were not long enough. In any case, long-term regular follow-up is essential for this disease.

The strength of our study is that to our knowledge, we are the first to report the incidence of histopathologically confirmed IgG4-RLD. Furthermore, we analyzed the clinicopathological characteristics of the patients with definite IgG4-RLD based on the histopathological diagnosis, and a brief diagnostic approach to distinguish IgG4-RLD from pulmonary cancer was suggested. However, the present study has some limitations. First, the generalizability of these findings may be questionable because our study is a retrospective single-center study with only a small number of cases and inherent selection bias. Large-scale prospective studies are needed for further confirmation. Second, our follow-up time was not sufficiently long, so the entire course of IgG4-RLD could not be well investigated.

## Conclusions

IgG4-RLD is a rare disorder that often mimics lung cancer. For clinicians, it is a great challenge to distinguish IgG4-RLD from lung cancer, especially when a single lung is affected or if the involvement of other organs does not provide diagnostic support. This study analyzed the clinical characteristics of 10 patients with pathologically confirmed IgG4-RLD in our hospital. Our results suggest that multiple imaging findings, elevated serum IgG4 level and no significant increase in serum tumor biomarkers could provide diagnostic support for IgG4-RLD, and subsequent experimental steroid therapy could be given to further confirm the diagnosis. If not, lung biopsy can be performed to make a definitive diagnosis.

## Data Availability

Data are available from the corresponding author upon request.

## Abbreviations

^18^F-FDG PET-CT: ^18^F-fluorodeoxyglucose positron emission tomography-computed tomography
CA125: Cancer antigen 125
CEA: Carcinoembryonic antigen
CT: Computed tomography
CYFRA 21-1: Cytokeratin fraction 21-1
ESR: Erythrocyte sedimentation rate
F: Female
GGO: Ground-glass opacity
H&E: Hematoxylin and eosin
HPF: High-power field
hs-CRP: High-sensitivity C-reactive protein
IgG: Immunoglobulin G
IgG4: Immunoglobulin G4
IgG4-RD: Immunoglobulin G4-related disease
IgG4-RLD: Immunoglobulin G4-related lung disease
M: Male
NA: Data not available
NSE: Neuron-specific enolase
OLB: Open lung biopsy
OOI: Other organ involvement
PLB: Percutaneous lung biopsy
TLB: Transbronchial lung biopsy
VATS: Video-assisted thoracoscopic surgery
